# DRANetSplicer: A Splice Site Prediction Model Based on Deep Residual Attention Networks

**DOI:** 10.3390/genes15040404

**Published:** 2024-03-26

**Authors:** Xueyan Liu, Hongyan Zhang, Ying Zeng, Xinghui Zhu, Lei Zhu, Jiahui Fu

**Affiliations:** 1 College of Information and Intelligence, Hunan Agricultural University, Changsha 410128, China; liuxueyan@stu.hunau.edu.cn (X.L.); zhuxh@hunau.edu.cn (X.Z.); leizhu@hunau.edu.cn (L.Z.); www.fjhsz2021@stu.hunau.edu.cn (J.F.); 2 School of Computer and Communication, Hunan Institute of Engineering, Xiangtan 411104, China; lexiaobao0503@sina.com

**Keywords:** splice site prediction, deep convolutional neural network, residual learning, attention mechanism

## Abstract

The precise identification of splice sites is essential for unraveling the structure and function of genes, constituting a pivotal step in the gene annotation process. In this study, we developed a novel deep learning model, DRANetSplicer, that integrates residual learning and attention mechanisms for enhanced accuracy in capturing the intricate features of splice sites. We constructed multiple datasets using the most recent versions of genomic data from three different organisms, *Oryza sativa japonica*, *Arabidopsis thaliana* and *Homo sapiens*. This approach allows us to train models with a richer set of high-quality data. DRANetSplicer outperformed benchmark methods on donor and acceptor splice site datasets, achieving an average accuracy of (96.57%, 95.82%) across the three organisms. Comparative analyses with benchmark methods, including SpliceFinder, Splice2Deep, Deep Splicer, EnsembleSplice, and DNABERT, revealed DRANetSplicer’s superior predictive performance, resulting in at least a (4.2%, 11.6%) relative reduction in average error rate. We utilized the DRANetSplicer model trained on *O. sativa japonica* data to predict splice sites in *A. thaliana*, achieving accuracies for donor and acceptor sites of (94.89%, 94.25%). These results indicate that DRANetSplicer possesses excellent cross-organism predictive capabilities, with its performance in cross-organism predictions even surpassing that of benchmark methods in non-cross-organism predictions. Cross-organism validation showcased DRANetSplicer’s excellence in predicting splice sites across similar organisms, supporting its applicability in gene annotation for understudied organisms. We employed multiple methods to visualize the decision-making process of the model. The visualization results indicate that DRANetSplicer can learn and interpret well-known biological features, further validating its overall performance. Our study systematically examined and confirmed the predictive ability of DRANetSplicer from various levels and perspectives, indicating that its practical application in gene annotation is justified.

## 1. Introduction

In recent years, the development of gene sequencing technologies has provided us with essential raw genomic data for studying and understanding biological organisms and their mechanisms. The raw genomic sequences generated by gene sequencing are often challenging to be directly utilized in research and require genomic annotation to be made accessible to relevant researchers. The progress of research endeavors, such as whole-genome analysis and differential gene expression analysis, heavily relies on accurate genomic annotation. In the gene structure of eukaryotic organisms, gene sequences exhibit a discontinuous nature, with coding regions (exons) and non-coding regions (introns) interspersed [1]. An important step in genomic annotation is the precise identification of the boundaries between exons and introns within the gene structure [2]. The boundaries between exons and introns are termed splice sites, with the donor splice site situated at the $5^{\prime }$ start of the intron (i.e., the exon-intron boundary) and the acceptor splice site located at the $3^{\prime }$ end of the intron (i.e., the intron-exon boundary) [3]. Within eukaryotic genomes, splice sites are highly conserved, with nearly 99% of splice sites conforming to the GT-AG nucleotide pattern, denoted as canonical sites [4]. However, some splice sites within the genome do not adhere to the GT-AG pattern, and these are referred to as non-canonical sites [5]. Accurately identifying splice sites is an essential pathway for precisely demarcating the boundaries between coding and non-coding regions, representing an important step in understanding gene structure and ensuring the accurate annotation of genomic sequencing data [6]. The prediction of splice sites is typically treated as a binary classification problem, where the results are categorized into true splice sites and false splice sites. Since splice sites are divided into donor splice sites and acceptor splice sites, and these two types of splice sites exhibit substantial differences, the prediction of both types is usually regarded as two different binary classification problems.

Existing splice site prediction methods can usually be categorized into three classes: probabilistic models, traditional machine learning approaches, and deep learning methods. Probabilistic models played an important role in the early stages of splice site prediction by effectively capturing sequence patterns and statistical features of splice sites. They provided a framework for understanding the probability distribution of splice events, laying the foundation for subsequent research [7]. Traditional machine learning methods contributed significantly, especially on moderate-sized datasets by incorporating additional feature engineering. Through the manual extraction of sequence features, these methods better explained the structure and patterns of gene sequences, offering an intuitive understanding of splice site prediction problems [8]. Deep learning methods achieved remarkable breakthroughs in the field of splice site prediction. Leveraging end-to-end learning with neural networks these methods autonomously learned complex features of gene sequences without extensive manual intervention. Deep learning methods demonstrated outstanding performance on large-scale datasets, particularly excelling in handling nonlinear and highly abstract biological information [9]. With the ongoing development of deep learning technologies, these methods have become mainstream in the field of splice site prediction.

On the other hand, existing deep learning methods have predominantly used relatively shallow network architectures for splice site prediction [10,11]. Numerous studies have demonstrated that the depth of a neural network is important for improving model performance [12,13]. However, deep neural networks can suffer from the issue of degradation as the network depth increases. To address this problem and optimize the training of deep networks, Kaiming He et al. introduced the concept of residual network (ResNet) structures [14]. More recently, Qilong Wang et al. proposed an efficient channel attention module called the Efficient Channel Attention (ECA) module, specifically designed for deep convolutional neural networks (CNN) [15]. This module effectively captures inter-channel interactions, allowing the network to focus more on critical features and suppress less important ones. This enhancement contributes to improved feature representation and generalization capabilities of the model. In this study, we incorporated the ECA module into the original residual learning framework, resulting in Residual Attention Modules (RAM). This module addresses the problem of model degradation caused by increased network depth and enhances the predictive performance of the network. We made improvements to the ResNet18 model [14] by replacing the original residual modules with our RAM and enhancing the downsampling mechanism to preserve genetic sequence information. Additionally, we employed larger convolutional kernels and reduced the convolutional stride to increase the model’s receptive field for capturing global features of genetic sequences. The model developed in this way is referred to as DRANetSplicer.

For our experiments, we selected three extensively studied model organisms: *O. sativa japonica*, *A. thaliana* and *H. sapiens*. We constructed corresponding whole-genome datasets using their respective latest gene annotation versions. This approach enables us to employ a more extensive set of high-quality data for training our models, thus enhancing their predictive performance. We validated the robustness of DRANetSplicer on splice site datasets from the selected organisms, obtaining consistent experimental results. Compared to benchmark methods, we demonstrate that DRANetSplicer achieves superior predictive performance. Through cross-organism validation, we establish the model’s high generalization capability, indicating its potential for splice site prediction across different organisms. We introduce a variety of visualization methods to more intuitively display the learned features of DRANetSplicer, which can also further validate the performance of the model.

## 2. Related Work

Drawing insights from previous studies on constructing splice site datasets, we observe the following key points. Donor and acceptor splice sites represent two distinct types of splice sites, each characterized by significant differences in contextual sequence features [16]. The model achieved optimal predictive performance when extracting 200 nucleotides from the upstream and downstream flanking regions of the splice site as input sequences [17]. Non-canonical splice sites are important in certain biological events, and neglecting their presence may lead to false negatives in identification results [18]. The strong conservation of splice sites does not effectively identify them as the GT-AG di-nucleotide, characteristic of splice sites, can also occur in non-splice site regions, resulting in higher false positives in predictions [17]. Training models on imbalanced datasets, where one class is overly represented, may lead to overfitting, making the model more prone to biased outcomes [19]. Furthermore, numerous studies on genome function prediction underscore the importance of establishing balanced datasets [20,21].

Early splice site prediction methods were primarily based on probabilistic models, such as the use of generalized hidden Markov models [7] and Markov models [22]. Since probabilistic models typically need to account for long-range dependencies in sequences, this may result in higher model complexity, leading to relatively larger computational costs in both training and inference processes. With the advancement of machine learning, it became possible to construct high-complexity models, and the use of machine learning methods to build classifiers for splice site prediction gradually became the mainstream approach. Machine learning-based splice site prediction methods typically involve manual feature extraction and machine learning model selection. Feature extraction is commonly based on biological characteristics such as base positions, correlations between adjacent or non-adjacent nucleotides, RNA secondary structures, and other relevant factors for representing gene sequences. Machine learning models use these artificially constructed features as model inputs, which are then employed to predict splice sites. Commonly used machine learning models include Support Vector Machines (SVM) [23,24], Artificial Neural Networks (NN) [25], Random Forests (RF) [26,27], Decision Trees (DT) [28], and Naïve Bayes (NB) [29]. While these machine learning methods have achieved significant success in splice site prediction tasks, the limitation lies in the inability of manual feature extraction to capture more significant distinctions between sequences, resulting in the predictive performance of machine learning models not reaching higher levels.

In recent years, deep learning methods have gained widespread adoption in splice site prediction. Models using deep learning techniques such as SpliceRover [9], Splice2Deep [30], Deep Splicer [10], EnsembleSplice [11], SpliceFinder [17], Spliceator [16], DeepSS [31], iSS-CN [32], DNABERT [33], and others have been developed for splice site prediction. The predictive performance of these deep learning models consistently outperforms traditional machine learning models. These models employing deep learning methods are all based on CNN architectures. CNN architectures can autonomously perform feature extraction, which addresses the limitations associated with manual feature extraction. However, these models have typically utilized relatively shallow network structures for splice site prediction. Numerous studies have emphasized the critical role of network depth in enhancing model performance. Using deeper network architectures can effectively elevate performance [12,13].

Deep neural networks are notoriously challenging to train, often leading to the problem of model degradation. In an effort to optimize the training process for deep networks, Kaiming He et al. introduced the ResNet framework [14]. They demonstrated that ResNet is easier to optimize and can achieve improved accuracy with significant depth. ResNet was initially designed for image processing tasks, which involve non-sequential data. Therefore, the original ResNet architecture was not explicitly designed to capture dependencies between elements. However, gene sequences are inherently sequential data, and the relationships between sequence elements play an important role in model predictions. The original ResNet applies downsampling in the initial stages and globally employs small convolutional kernels and large strides to reduce computational complexity. This approach can result in a significant loss of genetic information within gene sequences and an inability to capture dependencies between functional regions in the gene.

In the past several years, residual networks have been widely applied in the field of bioinformatics, and experts and scholars in this field have increasingly valued the role of residual networks, achieving good performance in bioinformatics tasks. Lijuan Shi et al. proposed the ResnetAge method, which utilizes deep learning techniques, particularly leveraging the advantages of ResNet to extract features from DNA methylation data and build models for predicting the biological age of individuals, providing a new solution for age prediction research based on DNA methylation data [34]. Mobeen Ur Rehman et al. introduced the DCNN-4mC method, where the residual connections incorporated in DCNN-4mC allow the sharing of shallow features with deeper layers, significantly improving the accuracy and robustness of the model in predicting N4-methylcytosine sites [35]. Muhao Chen et al. proposed a model called PIPR, which integrates a deep residual recurrent convolutional neural network into a Siamese architecture. This model models protein sequences to predict protein-protein interactions, and experimental results demonstrate that PIPR outperforms various state-of-the-art systems [36]. Panagiotis Korfiatis et al. proposed a method for predicting the methylation status of the MGMT gene using a residual deep convolutional neural network. By introducing residual connections into the deep convolutional neural network, the model’s performance and training speed are improved, enabling prediction of the methylation status of the MGMT gene, which contributes to cancer diagnosis and treatment research [37].

Qilong Wang et al. introduced an efficient channel attention module (ECA) designed specifically for deep CNN [15]. This module is highly effective in enhancing the performance of deep CNN by capturing cross-channel interactions. The core idea behind ECA is to introduce a channel attention mechanism within the convolution operation to capture relationships between different channels, thus improving the capability of feature representation. The goal of the channel attention mechanism is to adaptively adjust the weights of channel features, allowing the network to focus more on important features while suppressing less relevant ones. This significantly enhances the model’s ability to generalize. Through this mechanism, ECA can effectively augment the network’s representational power without introducing excessive parameters or computational costs.

Kishore Jaganathan et al. [38] and Johannes Linder et al. [39] have provided substantial evidence in their research, demonstrating that deep learning models can effectively learn known biological features. They suggest that future deep learning models hold the potential to offer insights into biology that human experts have not yet described, serving not only as black-box classifiers. Similarly, we validate DRANetSplicer’s performance by confirming its capacity to learn well-known biological features.

The most commonly used visualization methods for gene sequence prediction models include DeepLIFT [40] and Grad-CAM [41]. DeepLIFT calculates contribution scores by comparing the activation of each neuron with a reference activation. Grad-CAM utilizes pre-trained weights to backpropagate to the parameter layer to generate important heatmaps for visualization. Both methods are excellent visualization techniques proposed in recent years. Unfortunately, DeepLIFT cannot visualize residual networks. To address this limitation, we introduce an extension of DeepLIFT called DeepSHAP [42]. DeepSHAP has the same architecture as DeepLIFT and supports operations where DeepLIFT does not propose backpropagation rules. In summary, to visualize the features learned by the DeepNetSplicer model and explore its interpretability, we employed both DeepSHAP and Grad-CAM methods. To validate the model’s ability to learn biological features, we compare visualizations generated by the model with well-known biological gene motif patterns displayed using WebLogo [43]. WebLogo effectively calculates sequence conservation and nucleotide relative frequencies at each nucleotide position from a multiple sequence alignment of genomic datasets and further generates sequence logos that represent known gene motif patterns.

## 3. Materials and Methods

### 3.1. Datasets Construction

We used the genome sequences of *O. sativa japonica*, *A. thaliana* and *H. sapiens* in this experiment to construct donor and acceptor splice site datasets, respectively, totaling six datasets, as shown in Table 1. During the dataset construction process, due to the double-helix structure of DNA molecules that exhibit reverse complementarity, we only extracted splice sites from the forward strand for analysis. We developed Python scripts to extract splice sites and their surrounding nucleotide sequences from the FASTA files based on the annotation information in the GFF files to construct the dataset. Moreover, all datasets were constructed using the whole genome sequences corresponding to the respective organisms, thus containing not only splice sites from canonical transcripts but also all splice sites annotated in the GFF files. In this study, genomic sequence data (FASTA files) and their respective genome annotation files (GFF files) were downloaded from NCBI. These FASTA and GFF files, maintained by the NCBI, can be readily accessed on the NCBI official website using the NCBI RefSeq version numbers.

We primarily considered the following aspects when constructing the dataset, whether to construct datasets separately for donor and acceptor splice sites, input sequence length, inclusion of non-canonical splice sites, types of negative datasets, and the ratio of positive to negative samples. We constructed separate datasets for donor and acceptor splice sites. From the upstream and downstream flanking segments of the splice sites, we extracted 200 nucleotides each, resulting in input sequences of length 402 nucleotides. The dataset construction included consideration of non-canonical splice sites. For the negative class dataset, we selected non-splice site sequences that adhere to the GT-AG rule. Redundant sequences were removed from the dataset, and we ensured an equal number of positive and negative samples (i.e., an equal number of splice and non-splice site instances).

In this study, we conducted experiments on the six datasets we created, splitting each dataset into training, validation, and test sets in proportions of 60%, 15%, and 25%, respectively, for model training, validation, and testing.

### 3.2. The DRANetSplicer Model

The DRANetSplicer model uses the RAM we designed as building blocks. ResNet, initially designed for non-sequential image data, lacks optimization for gene sequence analysis due to its early downsampling and small convolutional kernels. To address this, we propose RAM, an improved building block for gene sequence prediction. (1) Delayed Downsampling: We postpone data downsampling until the third convolutional stage to preserve genetic information integrity; (2) Improved Downsampling: Instead of using stride-2 convolutions for downsampling, we first extract features with stride-1 convolutions, followed by downsampling using average pooling to avoid information loss; (3) Larger Receptive Field: We use larger convolutional kernels to capture broader features, essential for distinguishing splice sites and non-splice sites with critical information in upstream and downstream sequences; (4) Residual Attention Module (RAM): Enhances Residual Blocks with ECA for improved feature extraction and cross-channel interaction, boosting the model’s representational power. These modifications aim to optimize ResNet for gene sequence analysis, particularly in predicting splice sites with higher accuracy and robustness.

**Figure 1:**
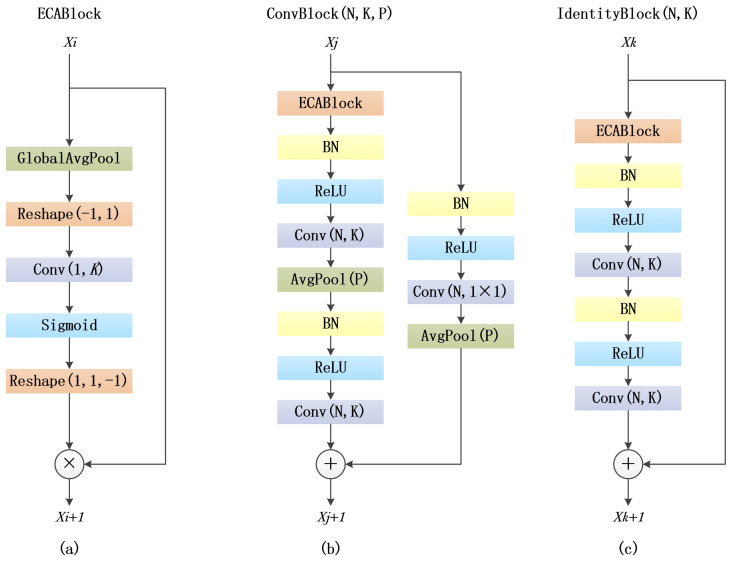
The structure of the RAM. (**a**) ECABlock represents the structure of the ECA. (**b**) ConvBlock illustrates the one of RAM. (**c**) IdentityBlock illustrates the other of RAM.

The structure of the RAM is shown in Figure 1. Figure 1a illustrates the ECABlock, which is the structure of the ECA. Figure 1b shows the ConvBlock, while Figure 1c displays the IdentityBlock, both representing the structure of our designed RAM. We constructed two RAMs to address whether changes occur in the size and dimensions of input and output feature maps. The difference between ConvBlock and IdentityBlock lies in the fact that the input and output feature maps in the IdentityBlock have the same size and dimensions, enabling them to be directly added together. However, in the ConvBlock structure, the output feature map undergoes changes in size and dimensions. In this case, a 1 × 1 the convolutional layer is applied, along with average pooling, for downsampling to match the dimensions and size of the input and output feature maps.

The ECABlock aggregates convolutional features using global average pooling, adaptively determines the convolution kernel size, performs one-dimensional convolution, and employs the Sigmoid function to learn channel attention. The ConvBlock consists of ECABlock, a convolutional layer, a batch normalization layer, an activation layer (utilizing Rectified Linear Unit, ReLU), an average pooling layer, and an additive layer. The IdentityBlock includes ECABlock, a convolutional layer, a batch normalization layer, an activation layer (using ReLU), and an additive layer. In both ConvBlock and IdentityBlock, the convolutional layer has a stride of 1. The hyperparameters N, K, and P on the convolutional layer and the average pooling layer represent the number of kernels, kernel size, and pooling window size, respectively. Before each convolutional layer in the ConvBlock and IdentityBlock, Batch Normalization is applied to accelerate training and reduce overfitting. It normalizes the input data for each batch, making the model more robust to variations in input data, which helps the model generalize better to new data.

**Figure 2:**
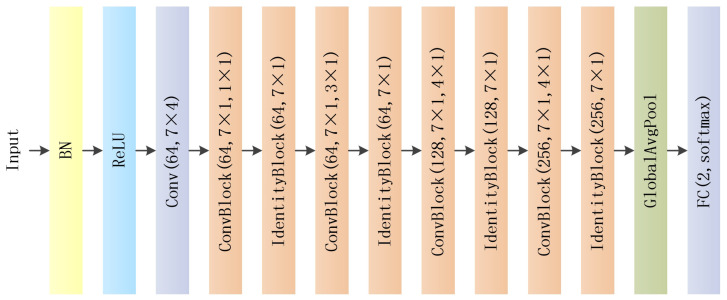
Depicts the architecture of DRANetSplicer.

The overall structure of DRANetSplicer is illustrated in Figure 2. The overall architecture of DRANetSplicer is an improvement upon the standard ResNet18 framework. In Section 4.2, we provide the rationale for selecting ResNet18 as the main backbone structure. We replace the original residual blocks in ResNet18 with RAM and use a grid search algorithm to determine the optimal combination of hyperparameters N, K, and P within the RAM. In the experiments, for the input to the DRANetSplicer model, the input layer receives a specific-sized one-hot encoded sequence. The input data first pass through a convolutional layer with 64 convolutional kernels, each with a size of 7 × 4, followed by batch normalization and an activation layer using Rectified Linear Unit (ReLU) as the activation function. Subsequently, it goes through four sets of ConvBlock and IdentityBlock with 64, 128, 128, and 256 convolutional kernels, all having a size of 7 × 1. The average pooling layer is applied with pooling window sizes of 1 × 1, 3 × 1, 4 × 1, and 4 × 1, respectively. The network concludes with a global average pooling layer and a two-way fully connected layer with Softmax. The final fully connected layer with two units corresponds to the two output classes activated by the Softmax function (splice sites and non-splice sites).

Table 2 illustrates the search space for hyperparameter optimization of DRANetSplicer. The best-performing hyperparameter combination, highlighted in bold is ultimately employed as the parameter for the DRANetSplicer model. In this study, we used an optimized grid search algorithm that tailors specific search spaces for each block instead of exhaustively searching all predefined parameter combinations. We formulated different hyperparameter search spaces for each block based on the five convolutional stages of ResNet. Here is a summary of the algorithm’s steps: (1) Define parameter ranges: Define possible ranges for each RAM hyperparameter (N, K, P) based on prior knowledge and computational resources; (2) Create parameter grids: Create parameter grids for each RAM based on the defined ranges; (3) Model training and evaluation: Train and evaluate the model using cross-validation on the training set, with accuracy as the evaluation metric; (4) Select the best combination: Choose the parameter combination with the best performance on the validation set as the final hyperparameters. This approach optimizes the search process by considering specific block features, leading to improved model performance.

### 3.3. Model Inputs and Outputs

**Figure 3:**
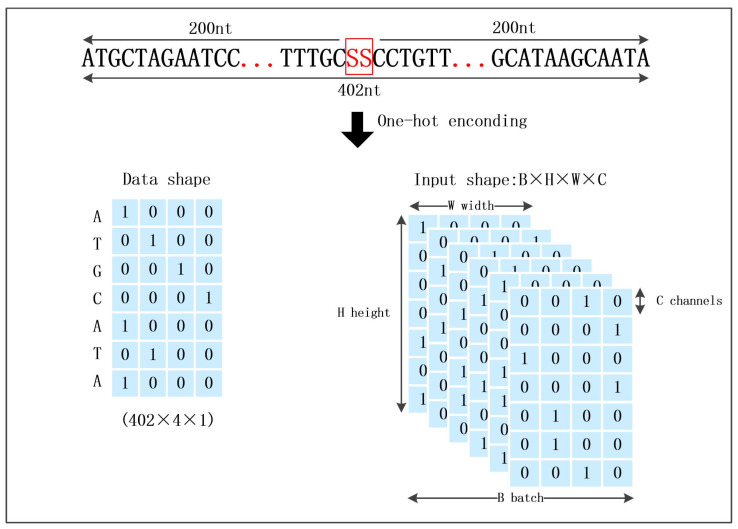
Model inputs. Nucleotide sequences are represented using one-hot encoding. SS represents splice sites, which include not only the canonical GT-AG sites but also non-canonical sites.

We converted nucleotide sequences into numerical vectors using one-hot encoding, where A, T, G, and C were, respectively, transformed into [1,0,0,0], [0,1,0,0], [0,0,1,0], and [0,0,0,1], as illustrated in Figure 3. Output variables were also one-hot encoded: splice sites and non-splice sites were mapped to [1,0] and [0,1], respectively.

### 3.4. Implementation Details

During the training process, we employed categorical cross-entropy as the loss function, Stochastic Gradient Descent (SGD) as the optimizer, and enabled Nesterov momentum for SGD. The initial learning rate was set to 0.01, and in the subsequent epochs, we reduced the learning rate by half every five epochs, continuing until 20 epochs of training. The batch size was set to 64. The model with the best performance on the validation set was selected as the final model. The training was repeated three times for each model individually and the average of the three results was used as the final result. All experiments were implemented using Tensorflow (version 2.6.0) and Keras (version 2.6.0) on the same system with a single NVIDIA GA102 [GeForce RTX 3090 Ti] 24 GB GPU.

### 3.5. Evaluation Metrics

For this experiment, six metrics were used to evaluate the model’s performance, and the calculation of these metrics is presented in Table 3. Accuracy represents the proportion of correctly identified samples to the total number of samples. Precision is the proportion of samples correctly identified as splice sites that are true splice sites. Sensitivity measures the proportion of correctly recognized splice site samples to the total number of splice site samples. Specificity represents the proportion of samples correctly identified as non-splice sites among the total number of non-splice site samples. The F1 score is the harmonic mean of precision and sensitivity, indicating the balance between these two metrics.

### 3.6. Cross-Organism Validation

Most splice site prediction models are trained on high-quality, experimentally verified data, which are predominantly available for well-studied organisms. However, for less-researched species, collecting high-quality datasets can be challenging. Since splice sites and other functional elements may exhibit conservation across similar species [44], some studies have explored the transfer of models trained on one species to similar species [19,45], such as within animals or plants. In line with the concept of cross-organism models, we estimate the generalization capability of DRANetSplicer for predicting splice sites in different species using cross-organism validation. Cross-organism validation involves testing a model trained on data from one species on data from another species. For example, cross-organism validation entails testing a model trained on *Oryza* data on data from two other species (*Arabidopsis* and *Homo*).

### 3.7. Interpretability

DeepSHAP allows the calculation of separate contribution scores for the four bases at each nucleotide position. The algorithm interprets the difference between the output results and the results under some “reference” input. It decomposes this output “reference difference” into the “reference difference” contributions of input features. Finally, contribution scores are obtained based on the designed operational rules.

WebLogo is utilized to efficiently compute the sequence conservation at each nucleotide position and the relative frequency of nucleotides from a multiple sequence alignment of genomic datasets. Using these computed data, sequence logos are generated, providing well-known gene motif patterns. We employed WebLogo to calculate the sequence conservation in the positive donor and acceptor datasets. These results were then compared with the nucleotide-weighted contribution scores learned by our model, serving as a validation of the model’s ability to learn known gene sequence motifs.

Grad-CAM operates by utilizing pre-trained weights during classification to perform backpropagation to the desired parameter layer (such as the convolutional layer, visualizing the final convolutional layer in this study). This process yields a gradient matrix for the output feature maps of that parameter layer. The gradient matrix is subjected to spatial dimension global average pooling, resulting in a weight vector for the output feature map channels. This weight vector is then used to weigh the various channels of the feature map, ultimately producing a heatmap.

To analyze the sequence features learned by the model, we conducted feature visualization on six different datasets: *Oryza* donor, *Oryza* acceptor, *Arabidopsis* donor, *Arabidopsis* acceptor, *Homo* donor, and *Homo* acceptor. As we need to compare the visualization results obtained from the above datasets, we employed the same approach to select 1000 input sequences from the test set for model visualization. However, there is a consideration: the contribution scores computed by DeepSHAP represent the contributions of all nucleotides in each input sequence within a given dataset. This leads to the issue that the contribution scores are not calculated on the same scale. Therefore, we adopted the normalization method used by Jasper Zuallaert et al. [9] to solve this problem and obtain the weighted contribution scores for the given dataset.

## 4. Results and Discussion

### 4.1. Prediction Performance of DRANetSplicer

To evaluate the performance of DRANetSplicer, we computed the six performance metrics as presented in Table 3. In addition, we provided the Area Under the ROC Curve (AUC). The predictive results of DRANetSplicer for three different organisms are shown in Table 4, please see Supplementary Materials Table S1 for the results of each individual experiment.

On the donor splice site dataset, DRANetSplicer achieved an average accuracy of 96.57%, an average error rate of 3.43%, and an average AUC of 99.05%. The average values of precision, sensitivity, specificity, and F1 score metrics all exceeded 96%. On the acceptor splice site dataset, DRANetSplicer obtained an average accuracy of 95.82%, an average error rate of 4.18%, and an average AUC of 98.86%. The average values of precision, sensitivity, specificity, and F1 score metrics all exceeded 95%. These performance metrics indicate that DRANetSplicer exhibits excellent predictive performance across datasets from different species.

### 4.2. Ablation Study

The ablation experiment results are presented in Table 5, showing that as the model depth increases from ResNet18 to ResNet34 and ResNet50, the predictive performance of the models decreases. This is attributed to the deeper network structures having more parameters, while the gene sequence input data are insufficient to support learning with such a large number of parameters, leading to model overfitting. Therefore, we chose to improve upon ResNet18 to build DRANetSplicer.

DRANetSplicer is designed based on the ResNet18 architecture. To demonstrate the effectiveness of the improvements we made for gene sequence characteristics, we conducted ablation experiments on DRANetSplicer and compared it to the ResNet18 model. The experimental results are presented in Table 5, showing that DRANetSplicer outperforms ResNet18 significantly in terms of accuracy on datasets from three different species. Supplementary Materials Table S2 provides the model accuracy for each individual experiment. Furthermore, DRANetSplicer achieves substantial optimizations in terms of model Params and FLOPs compared to ResNet18, which results in reduced model size and improved memory/time efficiency.

### 4.3. Comparison with Benchmark Methods

To provide a more comprehensive evaluation of DRANetSplicer’s performance, we compared it with other state-of-the-art prediction models, including four outstanding deep learning methods: SpliceFinder [17], Splice2Deep [30], Deep Splicer [10], EnsembleSplice [11], and DNABERT [33].

As shown in Table 6, the DRANetSplicer model outperforms the benchmark methods in terms of accuracy and F1-score on both the donor and acceptor datasets for all three biological species. A comparison of the performance of each individual experiment with the benchmark method is shown in Supplementary Materials Table S3. In the donor splice site dataset, DRANetSplicer achieves the best average accuracy of 96.57%, resulting in average error rate relative reductions of 28.4%, 58.8%, 48.1%, 19.1%, and 4.2% compared to SpliceFinder, Splice2Deep, Deep Splicer, EnsembleSplice, and DNABERT, respectively. In the acceptor splice site dataset, DRANetSplicer attains the highest average accuracy of 95.82%, resulting in average error rate relative reductions of 39.2%, 58.1%, 64.4%, 23.7%, and 11.6% compared to the same benchmark models. Across both donor and acceptor splice sites, DRANetSplicer consistently reduces the relative error rate, with at least a 4.2% and 11.6% relative reduction in average error rates compared to the benchmark models.

**Figure 4:**
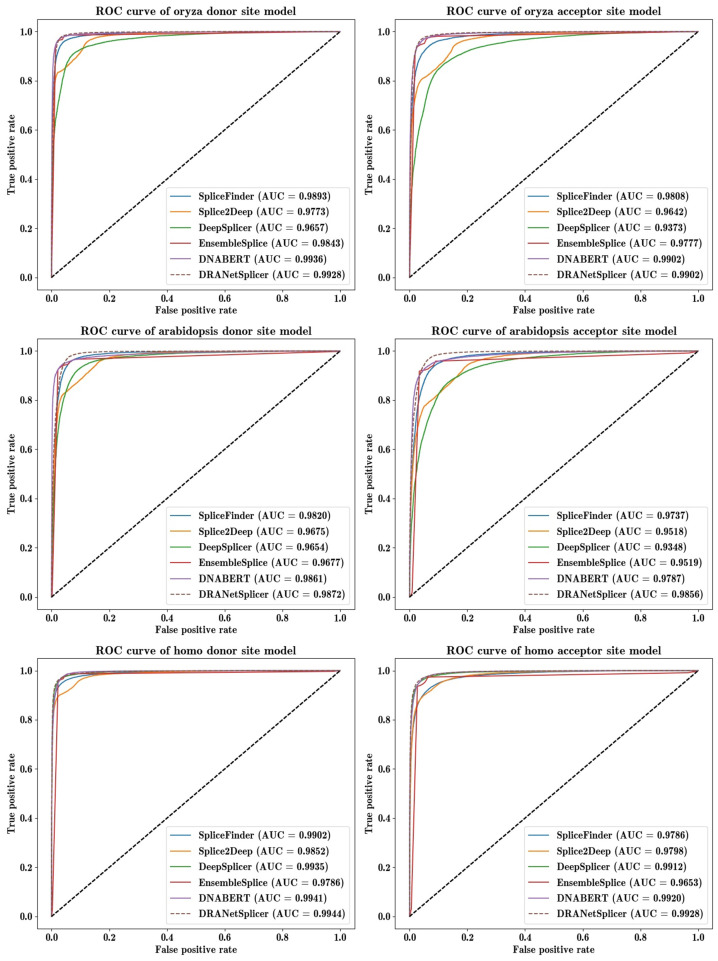
ROC curve of donor site model and acceptor site model.

In Table 6, we also include the harmonic mean of sensitivity and specificity, which is the F1-score, reflecting the overall performance in correctly predicting splice sites. DRANetSplicer achieves an average F1-score of 96.54% for donor splice sites and 95.80% for acceptor splice sites, whereas the second-ranking model, DNABERT, achieves average F1-scores of 96.42% and 95.27%, respectively. This means that the average F1-score error (1 − F1-score) is relatively reduced by 3.4% and 11.2% for donor and acceptor splice sites, respectively. Additionally, we have plotted the ROC curves for DRANetSplicer and other benchmark models, as shown in Figure 4. From the curves, it can be observed that the performance of the DRANetSplicer model is excellent.

Importantly, in comparison to DNABERT, which represents the outstanding gene prediction model pretrained on DNA sequences with splice site prediction as one of its downstream tasks, DRANetSplicer significantly reduces the model’s parameter and computational complexity while ensuring predictive performance. Specifically, DNABERT has 99.95 M parameters (Params) and requires 2.56 G of computation (FLOPs), whereas DRANetSplicer has 2.66 M parameters and requires 0.34 G of computation. This makes DRANetSplicer more efficient than DNABERT and more suitable for deployment in production environments.

### 4.4. Cross-Organism Validation

The results of cross-organism validation are shown in Table 7. Experimental results for each cross-organism validation are provided in Supplementary Materials Table S4. When tested on *Oryza*, the accuracy of DRANetSplicer for both donor and acceptor sites trained on *Oryza* and *Arabidopsis* is (97.21%, 96.30%) and (93.58%, 93.40%), respectively. When tested on *Arabidopsis*, the accuracy for donor and acceptor sites trained on *Arabidopsis* and *Oryza* is (95.63%, 94.91%) and (94.89%, 94.25%), respectively. When tested on *Homo*, the accuracy for donor and acceptor sites trained on *Oryza* and *Arabidopsis* is (82.95%, 80.17%) and (78.00%, 75.99%), respectively. We observe that cross-organism validation between *Oryza* and *Arabidopsis* yields better results, while cross-organism validation between *Homo* and the other two species is less effective.

Combining the experimental results from Table 6, we notice a phenomenon where DRANetSplicer, when trained on one species and tested on another (cross-organism validation), achieves higher accuracy compared to the non-cross-organism validation of benchmark models. For instance, for donor sites, DRANetSplicer trained on *Oryza* and tested on *Arabidopsis* achieves an accuracy of 94.89%, whereas SpliceFinder, Splice2Deep, Deep Splicer, and EnsembleSplice trained and tested on *Arabidopsis* achieve accuracies of 94.43%, 88.91%, 91.42%, and 94.43%, respectively. Bold data in Table 7 indicate that the cross-organism predictions of DRANetSplicer have higher accuracy than the non-cross-organism predictions of benchmark models. The analysis suggests that DRANetSplicer exhibits strong generalization capabilities, particularly in cross-organism validation between similar organisms. The cross-organism validation between *Oryza* and *Arabidopsis* demonstrates superior results compared to cross-organism validation on *Homo* and the other two species.

Based on these findings, we conclude that DRANetSplicer is applicable to a broader range of organisms and can transfer well from one species dataset to related species with limited research. Therefore, the DRANetSplicer model is highly valuable for genome annotation work on less-studied organisms.

### 4.5. Interpretability

**Figure 5:**
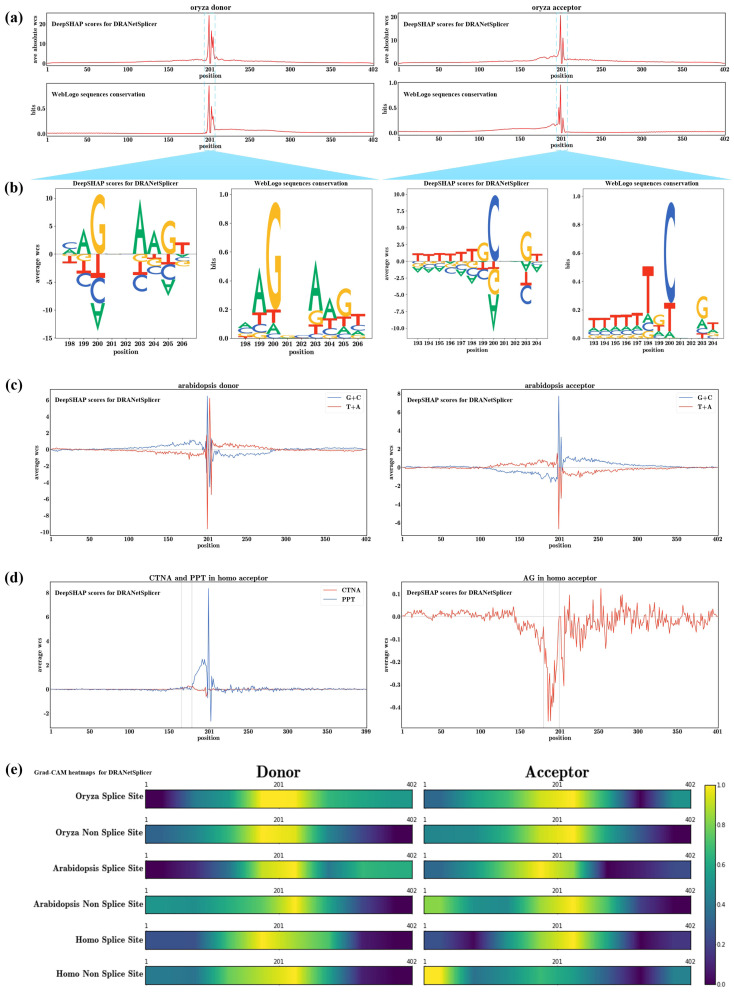
Explanatory analysis of the DRANetSplicer model. (**a**) Importance at each nucleotide position. We computed the average absolute weighted contribution scores for each nucleotide position in the *O. sativa japonica* donor and acceptor models, where donor and acceptor splice sites are located in the sequence’s 201–202 region. (**b**) Average weighted contribution scores for each of the four nucleotides at each position. For comparison, we utilized WebLogo to visualize known gene motifs. (**c**) Average weighted contribution scores for G + C and T + A in the donor and acceptor models of *A. thaliana*. (**d**) Average weighted contribution scores for the occurrence of CTNA and AG motifs in the *H. sapiens* acceptor model, where we denote the position of the motif by the position of the first nucleotide of the motif. (**e**) Heatmaps depicting the average nucleotide position importance calculated by the Grad-CAM method for the positive and negative datasets of the donor and acceptor models.

We computed the average absolute weighted contribution scores for each nucleotide position learned by the *Oryza* donor and acceptor models. The results were compared with the sequence conservation calculated by WebLogo for the positive dataset, as shown in Figure 5a. We observed a high similarity between the average absolute weighted contribution scores for each nucleotide position learned by the model and the sequence conservation at that position. The cosine similarities between the two in the *Oryza* donor and acceptor datasets were 0.93 and 0.90, respectively. This suggests that the model can effectively learn known gene motif patterns during the training process. Furthermore, the conclusion drawn from Figure 5a is that nucleotides around the splice sites have the most significant average impact on the model’s prediction results, and regions around the splice sites are generally more crucial than the marginal regions. The heatmap of importance for each nucleotide position learned by the donor and acceptor models, as shown in Figure 5e, confirms the correctness of this observation. This observation is consistent with the findings of previous studies by Jasper Zuallaert et al. [9] and Julie D. Thompson et al. [16].

In Figure 5b, we conducted a detailed investigation of the highlighted regions in the *Oryza* donor and acceptor models (marked in Figure 5a). We compared the individual average weighted contribution scores for the four nucleotides at each nucleotide position with the known gene motifs visualized by WebLogo. Within the studied regions, we observed that the most conserved nucleotide at each position, according to WebLogo, consistently corresponded to the nucleotide with the highest average weighted contribution score at that position in our model. Additionally, the proportions of average weighted contribution scores for different nucleotides at each position were similar to the proportions of nucleotide frequencies calculated by WebLogo.

According to the study by Amit et al. [46], the G + C content of exons in the *Arabidopsis* genome is significantly higher than that of introns. To validate whether our model learned this biological feature during training, we plotted the average weighted contribution scores for G + C and T + A at each nucleotide position in the *Arabidopsis* donor and acceptor models, as shown in Figure 5c. The plot illustrates that the upstream region (exons) of the donor splice site, indicated by positive average contribution scores for G + C and negative scores for T + A, exhibits the opposite trend in the downstream region (introns). Similarly, the downstream region (exons) of the acceptor splice site shows positive average contribution scores for G + C and negative scores for T + A, while the upstream region (introns) displays the opposite pattern. This analysis indicates that our model can automatically learn these biological features to distinguish between exons and introns.

In eukaryotic organisms, the splicing process is conserved, involving not only direct splicing at the donor and acceptor splice sites but also the participation of feature sequences such as branch points (BP) and polypyrimidine tracts (PPT). The PPT, rich in pyrimidine bases are located between the BP and the acceptor splice site, with the BP positioned several dozen nucleotides upstream of the PPT [47]. From Figure 5a, we observe that the *Oryza* acceptor model distinctly learns the PPT sequence features preceding the acceptor splice site, with the average weighted contribution scores for T + C in this region being positive. Iwata et al.’s study revealed a strong negative correlation between PPT and BP signals in eukaryotes, with a strong positive correlation with the acceptor splice site signal [47]. In Figure 5d, we investigate the impact of CTNA and PPT on splicing in the *Homo* acceptor model, where CTNA is a reported typical BP motif [47].

We observe a negative correlation between the average weighted contribution scores of CTNA and PPT in the region several dozen bases upstream of the acceptor splice site. Additionally, there is a small peak in the average weighted contribution scores of CTNA in a small region just before the rise in the average weighted contribution scores of PPT (highlighted in Figure 5d). Furthermore, we notice that as we approach the acceptor splice site, the average contribution scores of CTNA become smaller and negative, while PPT exhibit the opposite trend. These observations confirm that our model can automatically learn the biological features of BP and PPT.

Research by Gooding et al. suggests a unique biological feature upstream of the acceptor splice site, known as the AG exclusion zone [48]. To verify the model’s learning of this biological feature, we studied the average contribution scores of the AG motif in the *Homo* acceptor model, as shown in Figure 5d. We observe a sharp decrease and negative values in the average weighted contribution scores of the AG motif in a region just before the acceptor splice site.

Figure 5e displays the importance of heatmaps for each nucleotide position in the donor and acceptor models. In the heatmap, it is observable that nucleotides around splice sites have the greatest average impact on the model’s predictive results. Moreover, the broader regions around splice sites are generally more important than the marginal regions. The heatmaps of the three biological models mutually affirm the correctness of these observations, consistent with the findings in Figure 5a. Notably, we observe a high similarity between the heatmaps of *Oryza* and *Arabidopsis* models, indicating a high similarity in the nucleotide regions crucial for decision-making in the model learning process for these two organisms. However, there are substantial differences in the decisive nucleotide regions between the *Homo* model and the models of *Oryza* and *Arabidopsis*.

Our observation results from the nucleotide position heatmaps validate that the cross-species validation experiment results are accurate. The cross-species validation between *Oryza* and *Arabidopsis* performs better than the cross-organism validation involving *Homo*. This underscores that similar organisms have a high degree of similarity in the decision features learned during model training. From a model interpretation perspective, we further emphasize that DRANetSplicer possesses the capability for cross-species prediction of splice sites.

In summary, we have demonstrated that the DRANetSplicer model can learn known biological features, indicating its ability to automatically learn biological features from gene sequences. However, we need to further enhance model interpretability to achieve the goal of providing biological insights that human experts have not described yet, rather than being just a black-box classifier. This represents a major challenge and is a direction for our future work.

The numerical data used in all figures are included in Supplementary Materials Table S5.

## 5. Conclusions

We have developed DRANetSplicer, a splice site prediction model with a deeper network structure based on residual learning and attention mechanisms. Extensive evaluations, including model comparisons and cross-organism validation on splice site datasets from *O. sativa japonica*, *A. thaliana*, and *H. sapiens*, confirm the exceptional performance of DRANetSplicer. Not only does DRANetSplicer outperform other advanced deep learning models in splice site prediction, but it also contributes to genome annotation efforts for understudied organisms. Our visualizations consistently produced qualitatively coherent results, which were able to confirm known biological features. This indicates that DRANetSplicer possesses a high level of feature representation and discriminative ability during training, which is an important aspect for ensuring its predictive performance. We have thoroughly validated the exceptional performance of DRANetSplicer from multiple levels and perspectives, and it is feasible to apply the model in real production environments.

## Figures and Tables

**Table 1:** Description of gene annotation versions and the number of positive and negative samples used for each organism.

Species	Reference	Annotation	Dataset	Num. of Positive	Num. of Negative
*O. sativa japonica*	IRGSP-1.0	NCBI-RefSeq: GCF_001433935.1	Donor	72,013	72,013
			Acceptor	73,214	73,214
*A. thaliana*	TAIR10.1	NCBI-RefSeq: GCF_000001735.4	Donor	65,161	65,161
			Acceptor	65,505	65,505
*H. sapiens*	GRCh38.p14	NCBI-RefSeq: GCF_000001405.40	Donor	141,499	141,499
			Acceptor	137,455	137,455

**Table 2:** Search space for DRANetSplicer hyperparameter optimization.

Convolution Stage	Model Hyperparameters	Search Space
Stage 1	N	[16,32,**64**]
	K	[1 × 4,3 × 4,4 × 4,5 × 4,**7 × 4**]
Stage 2	N	[32,**64**,128]
	K	[5 × 1,**7 × 1**,9 × 1]
	P	[**1 × 1**,2 × 1,3 × 1]
Stage 3	N	[**64**,128,256]
	K	[5 × 1,**7 × 1**,**9 × 1**]
	P	[**3 × 1**,4 × 1,5 × 1]
Stage 4	N	[64,**128**,256]
	K	[**7 × 1**,9 × 1,11 × 1]
	P	[**3 × 1**,4 × 1,5 × 1]
Stage 5	N	[128,**256**,512]
	K	[**7 × 1**,9 × 1,11 × 1]
	P	[3 × 1,**4 × 1**,5 × 1]
Other hyperparameter	Pooling	[MaxPooling,**AveragePooling**]
	Optimizers	[Adam,Adamax,Nadam,**SGD**]
	Batch size	[16,32,**64**,128,256]
	Epochs	[10,**20**,30,40,50]

**Table 3:** Performance metrics used for model evaluation.

Evaluation Metrics	Equation
Accuracy (Acc)	$\frac{\mathrm{TP}\;+\;\mathrm{TN}}{\mathrm{TP}\;+\;\mathrm{TN}\;+\;\mathrm{FP}\;+\;\mathrm{FN}}$
Precision (Pre)	$\frac{\mathrm{TP}}{\mathrm{TP}\;+\;\mathrm{FP}}$
Sensitivity (Sn)	$\frac{\mathrm{TP}}{\mathrm{TP}\;+\;\mathrm{FN}}$
Specificity (Sp)	$\frac{\mathrm{TN}}{\mathrm{TN}\;+\;\mathrm{FP}}$
F1 Score (F1)	$\frac{2\times \mathrm{TP}}{2\times \mathrm{TP}\;+\;\mathrm{FP}\;+\;\mathrm{FN}}$
Error Rate(Err)	1− Accuracy

TP (True Positive): The number of splice sites correctly predicted as splice sites. FN (False Negative): The number of splice sites incorrectly predicted as non-splice sites. FP (False Positive): The number of non-splice sites incorrectly predicted as splice sites. TN (True Negative): The number of non-splice sites correctly predicted as non-splice sites.

**Table 4:** Performance metrics of DRANetSplicer in predicting donor and acceptor splice sites for three organisms.

Species	Site	Acc (%)	Pre (%)	Sn (%)	Sp (%)	Err (%)	F1 (%)	AUC (%)
*O. sativa japonica*	Donor	97.21	97.53	96.85	97.57	2.79	97.19	99.21
	Acceptor	96.30	96.29	96.29	96.32	3.70	96.29	98.97
*A. thaliana*	Donor	95.63	96.75	94.41	96.85	4.37	95.56	98.59
	Acceptor	94.91	95.68	94.05	95.76	5.09	94.86	98.40
*H. sapiens*	Donor	96.88	96.78	96.98	96.77	3.12	96.88	99.35
	Acceptor	96.25	95.93	96.58	95.91	3.75	96.25	99.20
Average	Donor	96.57	97.02	96.08	97.06	3.43	96.54	99.05
	Acceptor	95.82	95.97	95.64	96.00	4.18	95.80	98.86

**Table 5:** Accuracy in model ablation experiments.

Model	*Oryza*		*Arabidopsis*		*Homo*		Params (M)	FLOPs (G)
	Donor (%)	Acceptor (%)	Donor (%)	Acceptor (%)	Donor (%)	Acceptor (%)		
ResNet18	96.30	94.86	94.79	93.53	96.54	95.61	11.19	0.41
ResNet34	96.26	94.51	94.78	93.24	96.34	95.30	21.31	0.86
ResNet50	95.48	93.39	94.02	92.13	95.72	93.89	23.59	0.93
DRANetSplicer	**97.21**	**96.30**	**95.63**	**94.91**	**96.88**	**96.25**	**2.66**	**0.34**

**Table 6:** Comparison of prediction results between DRANetSplicer and benchmark methods.

Model	Site	*Oryza*		*Arabidopsis*		*Homo*		Average	
		Acc (%)	F1 (%)	Acc (%)	F1 (%)	Acc (%)	F1 (%)	Acc (%)	F1 (%)
SpliceFinder	Donor	95.55	95.50	94.43	94.36	95.65	95.69	95.21	95.18
	Acceptor	93.73	93.74	92.65	92.66	92.97	92.97	93.12	93.12
Splice2Deep	Donor	91.96	91.99	88.91	89.06	94.13	94.14	91.67	91.73
	Acceptor	90.99	91.05	86.13	86.39	92.98	92.97	90.03	90.14
Deep Splicer	Donor	92.12	92.04	91.42	91.02	96.63	96.64	93.39	93.23
	Acceptor	85.11	84.19	84.20	82.59	95.49	95.44	88.27	87.41
EnsembleSplice	Donor	96.47	96.48	94.43	94.44	96.38	96.40	95.76	95.77
	Acceptor	94.94	94.95	93.60	93.62	95.01	95.02	94.52	94.53
DNABERT	Donor	97.14	97.14	95.32	95.31	96.80	96.80	96.42	96.42
	Acceptor	95.94	95.94	93.80	93.80	96.06	96.06	95.27	95.27
DRANetSplicer	Donor	**97.21**	**97.19**	**95.63**	**95.56**	**96.88**	**96.88**	**96.57**	**96.54**
	Acceptor	**96.30**	**96.29**	**94.91**	**94.86**	**96.25**	**96.25**	**95.82**	**95.80**

**Table 7:** Accuracy of DRANetSplicer in cross-organism validation.

	Test	Site	*Oryza* (%)	*Arabidopsis* (%)	*Homo* (%)
Train					
*Oryza*		Donor	97.21	**94.89**	82.95
		Acceptor	96.30	**94.25**	80.17
*Arabidopsis*		Donor	**93.58**	95.63	78.00
		Acceptor	**93.40**	94.91	75.99
*Homo*		Donor	**91.97**	**91.44**	96.88
		Acceptor	**87.45**	**87.23**	96.25

“Train” indicates training the model with the respective biological data, and “Test” indicates testing the model with the corresponding biological data. Bold data points indicate that the cross-organism predictions of DRANetSplicer outperform the non-cross-organism predictions of certain benchmark models.

## Data Availability

All code and data used in this manuscript can be found at https://github.com/XueyanLiu-creator/DRANetSplicer (accessed on 20 January 2024).

## Supplementary Materials

The following supporting information can be downloaded at https://www.mdpi.com/article/10.3390/genes15040404/s1, Table S1: DRANetSpllicer respective prediction performance per experiment; Table S2: Accuracy in model per ablation experiments; Table S3: Comparison of performance with the benchmark method in each individual experiment; Table S4: Experimental results for each cross-organism validation; Table S5: Plotting basic data information for Figure 5.

## Abbreviations

The following abbreviations are used in this manuscript:

DRANetSplicer	A splice site prediction model based on deep residual attention networks
ResNet	Residual Networks
ECA	Efficient Channel Attention
CNN	Convolutional Neural Networks
RAM	Residual Attention Modules
SVM	Support Vector Machines
NN	Artificial Neural Networks
RF	Random Forests
DT	Decision Trees
NB	Naïve Bayes
ReLU	Rectified Linear Unit
SGD	Stochastic Gradient Descent
Acc	Accuracy
Pre	Precision
Sn	Sensitivity
Sp	Specificity
F1	F1 Score
Err	Error
AUC	Area Under the ROC Curve
TP	True Positive
FN	False Negative
FP	False Positive
TN	True Negative
Params	Parameters
FLOPs	Floating Point Operations
BP	Branch Points
PPT	Polypyrimidine Tracts
